# Does *in vitro* selection of biocontrol agents guarantee success *in planta*? A study case of wheat protection against *Fusarium* seedling blight by soil bacteria

**DOI:** 10.1371/journal.pone.0225655

**Published:** 2019-12-05

**Authors:** Yoann Besset-Manzoni, Pierre Joly, Aline Brutel, Florence Gerin, Olivier Soudière, Thierry Langin, Claire Prigent-Combaret

**Affiliations:** 1 UMR Ecologie Microbienne, CNRS, INRA, VetAgro Sup, UCBL, Université de Lyon, Villeurbanne cedex, France; 2 Biovitis, Le Bourg, Saint Etienne-de-Chomeil, France; 3 INRA, UMR 1095 Génétique, Diversité et Ecophysiologie des Céréales, Clermont–Ferrand, France; Georg-August-Universitat Gottingen, GERMANY

## Abstract

Biological control is a great hope for reducing the overutilization of pesticides in agricultural soils. It often involves microorganisms or molecules produced by microorganisms that will be able to interact with either a plant or pathogens of this plant to reduce the growth of the pathogen and limit its negative impact on the host plant. When new biocontrol products are developed, strains were mostly selected based on their ability to inhibit a pathogen of interest under *in vitro* conditions *via* antagonistic effects. Strains with no *in vitro* effect are often discarded and not tested *in planta*. But is the *in vitro* selection of bacterial agents according to their antagonism activities towards a plant pathogen the best way to get effective biocontrol products? To answer this question, we used wheat and the fungal pathogen *Fusarium graminearum* as a study pathosystem model. A library of 205 soil bacteria was screened in 2 types of *in vitro* growth inhibition tests against *F*. *graminearum*, and in an *in planta* experiment. We find strains which do not have inhibition phenotypes *in vitro* but good efficacy *in planta*. Interestingly, some strains belong to species (*Microbacterium*, *Arthrobacter*, *Variovorax*) that are not known in the literature for their ability to protect plants against fungal pathogens. Thus, developing a biocontrol product against *F*. *graminearum* must be preferentially based on the direct screening of strains for their protective activity on wheat plants against fungal diseases, rather than on their *in vitro* antagonistic effects on fungal growth.

## Introduction

Since the Green revolution, the way crops are grown has seen profound change. The use of chemical compounds designed by humans to improve health and productivity of plants has strongly expanded. By improving the quality and the yield of plant productions, and controlling agricultural pests, chemical fertilizers and pesticides brought beneficial effects to society [1–2]. All these benefits allowed the worldwide population to expand. But since 1963, the negative impacts of pesticides on human health, wildlife and environment have been extensively reported [3–4]. Negative impacts on biotic components can occur at different levels from individual organisms to communities, affecting the balance of the whole ecosystem. The main cause of this is that less than 0.1% of the pesticides used for pest control reach their targets accurately [5–6], leaving the rest of the active substances in the ecosystems. This leads to a problematic emergence of resistance to chemical pesticides in the phytopathogens [7]. Methods have been developed to evaluate the impact of pesticides on the environment, and they allowed to show that pesticide applications must be reduced to limit human health problems, environmental contamination and reduced soil fertility [7–9]. Simultaneously, new ways of crop production grouped under the generic term Integrated Pest Management (IPM) have been developed to control pests in agriculture. One mechanism involved in IPM strategies consists of the use of natural predators, antagonists, competitors or pathogens of pest targets, known as biological control agents. The principle of biocontrol is to maintain the targeted pest at a threshold below the limit responsible for negative impacts on the plant. Indeed, some microorganisms from soils have been shown to have beneficial effects on plant health and growth [10–11]. The mechanisms involved are quite diverse: antibiosis through the production of antimicrobial compounds like cyclic lipopeptides, polyketides, or lytic enzymes [12], (ii) competition for resources [11, 13], (iii) competitive exclusion [13], and (iii) elicitation of plant induced systemic resistance [14]. In addition, if the microorganisms have the ability to enhance the bioavailability or assimilation of essential nutrients such as nitrogen or phosphorus by the plant, it can help the plant to better cope with pathogen attacks [11]. In the hope of finding highly active biocontrol bacteria, the number of studies reporting screening of bacterial libraries against pests have grown exponentially.

In the present work, the Wheat-*Fusarium graminearum* pathosystem was used as a model system. *F*. *graminearum* is a well-known pathogen of small grain cereals and particularly wheat, capable of inducing four types of diseases: damping-off, root-rot, crown-rot, and *Fusarium* head blight (FHB or scab). FHB is one of the main fungal diseases of wheat. The FHB species complex produces mycotoxins that cause quality and yield reductions, as well as human and animal health risks. Cultural and management practices can reduce but not completely control FHB epidemics [15]. Due to its life cycle, solutions for chemical control of *F*. *graminearum* are not very effective [16–18]. In the literature, some bacterial genera, such as *Bacillus* or *Pseudomonas* are often found as bacteria used for biocontrol of *F*. *graminearum* in wheat [19–23]. More rarely, other genera like *Stenotrophomas* [19], *Acinetobacter* and *Chryseobacterium* [24], *Streptomyces* and *Brevibacillus* [25] are explored. These bacteria are often derived from either the rhizosphere soil of wheat, or from ears or grains, especially in the case of targeting bacterial endophytes [21]. The methods of selection of these bacteria often rely on the ability of the strains to inhibit the *F*. *graminearum* mycelial growth under *in vitro* conditions. Effective bacteria inhibiting the fungal growth are then tested in plant disease protection trials in a greenhouse, or directly in the field, on large or small scales [22, 24]. In all the studies, screening steps allow elimination of bacteria that do not show antagonistic effects on *F*. *graminearum*, either by the inhibition of the fungal growth or reduction of mycotoxin concentrations. But moving from laboratory to field trials often leads to bad performances and inconsistent results.

Thus, the literature confirms that screening is essentially made firstly on *in vitro* inhibition tests against a plant pathogen of interest and that secondly, only the best bacteria are selected for plant protection experiments. But, does selecting the best bacteria *in vitro* guarantee success *in planta*? In order to answer this question, we performed for this study a classic selection of bacterial strains made from *in vitro* dual-culture tests against *F*. *graminearum*. We then classified the bacteria from our library in different categories of antagonist activities. A wide selection of bacterial candidates harboring high or low *in vitro* activities was then tested in a greenhouse disposal to confirm the first screening step. A particular focus was made on some bacterial genera that were not often tested in the literature as biocontrol agents, in order to evaluate their potential protective activity against *F*. *graminearum*.

## Materials and methods

### Constitution of the bacterial strain library

In order to access to a wide diversity of bacterial strains, the library was built from 4 different wheat-growing soils from 4 locations in France, i.e. La Côte Saint André (LCSA; silty loam luvisol, 45° 22′ 43″ N, 5° 16′ 02″ E, Isère, France) [26], the Limagne basin near Clermont-Ferrand (clay-rich soil vertisol, 45° 47′ 6″ N, 3° 11′ 11″ E, Clermont-Ferrand, France) [27], Trelins (45° 43' 42.1" N, 4° 01' 34.6" E, Loire, France) and Yzeure-sur-Creuse (46° 48' 39.8" N, 0° 49' 47.2" E, Indre-et-Loire, France). For each sampling site, 5 g of rhizosphere soils were mixed with 10 ml of a 0.8% (w/v) NaCl solution. Serial dilutions of soil suspensions were carried out until a dilution of 10^−9^ and 100 μL of each suspension was spread on 1/10-diluted TSA (Tryptone Soya Agar, Carl Roth, Karlsruhe, Germany) as described by Bachate et al. [28]. After 48 hours of incubation at 28°C, isolated colonies were transferred to new TSA Petri plates and purified at least two additional times. Pure isolates were then grown in TSB (Tryptone Soya Broth, Carl Roth, Karlsruhe, Germany) for 24 h at 28°C and then aliquoted and stored at -80°C in glycerol (at 40%).

### Strains—Taxonomic characterization

Isolates from the whole library were taxonomically characterized based on the sequencing of their housekeeping 16SrRNA encoding *rrs* gene. Genomic DNA from all bacteria was extracted using the NucleoSpin Microbial DNA kit (Ref—740235.50, Macherey-Nagel, Germany). The *rrs* gene was amplified with primers pA/pH [29] and sequenced. The sequences were aligned with Muscle [30]. The phylogenetic tree was inferred using Seaview and the Neighbor-Joining distance-based method with 1000 bootstraps. Other methods were implemented and gave similar tree topologies. The tree was represented using iTOL [31]. The European Nucleotide Archive (ENA) accession number for the 203 *rrs* sequences reported in this paper is ERP115553. Other accession numbers used in this work are KT380501.1 for *Thaumarchaeota archaeon* NAOA2, NZ_AWQY01000007.1 for *Bacillus amyloliquefaciens* AB42 and AJ278814.1 for *Pseudomonas kilonensis* F113.

### *In vitro* screening of the efficacy of bacterial strains to inhibit the growth of *Fusarium graminearum*

The highly virulent and toxin-producing French isolate *Fusarium graminearum MDC_Fg1* (throughout referred to as *Fusarium graminearum Fg1*) used throughout experimentations was provided by Dr. Thierry Langin (GDEC Joint Research Unit, INRA Center Auvergne—Rhône-Alpes, Clermont-Ferrand, France). This strain was isolated from naturally infected cereal grains in the North of France. *Fg1* is able to induce FHB as well as the *Fusarium* crown-rot and seedling blight. For spore production, the cultures were grown on liquid Mung Bean Broth (MBB), for five days at 26 C. Spore concentration in the suspension was adjusted to 10^6^ spores.mL^−1^.

### Growth inhibition test on solid medium

Dual-culture protocols were implemented to assess the potential effect of bacterial antagonism on *Fg1* mycelium growth on solid medium. All experiments were carried out in triplicates. First, each bacteria was deposited 2 cm apart from the center of PDA (Potato Dextrose Agar, Conda Pronadisa, Madrid, Spain) plates as a 2 cm-long line, from TSB cultures grown over 24 hours. After incubation at 28°C for 24 hours, a 6 mm-diameter agar with mycelium sampled from the leading edge of a 5 day-old culture of *F*. *graminearum MDC_Fg1* grown on PDA was placed on the center of the plate. PDA plates were inoculated only with the pathogen for fungal control. The plates were then placed at 28°C in the dark for 5 days before the measurement of an inhibition index according to Shi et al. [32] with some modifications. The efficacy of pathogen growth inhibition was calculated according to 2 measurements carried out on the same plate: the radius of fungal growth towards the site of bacterial growth, and the radius between the center of the plate and the limit of fungal growth towards the site free of bacteria. The ratio between these 2 values was calculated and compared to that of the positive control without bacteria. Growth inhibition of mycelium on solid medium (GIm) was then estimated (S1 Fig). When GIm is lower than 0 for a given strain, the number is replaced by 0, and the strain is considered as a non-inhibitory strain.

### Growth inhibition test in liquid medium

Supernatants of bacteria were used to assess their antagonism potential on *Fg1* spore germination and/or mycelium growth in broth, in a microplate test. All experiments were carried out in triplicate. The supernatant of each bacteria was prepared from a 1-day old TSB culture, centrifuged at 4500 rpm during 10 min and filtered at 0.2 μm. For each treatment, 100 μL of supernatant, 100 μL of PDB (Potato Dextrose Broth, Conda Pronadisa) and 50 μL of *Fg1* asexual spores (macroconidia) suspension at 10^4^ spores.mL^-1^ were added per microplate wells. As positive control, 100 μL of TSB were used to replace the bacterial supernatants. As negative control, 50 μL of PDB were used to replace the *Fg1* spore suspension. After 5 days of culture at 28 °C, the microplates were analyzed using an Infinite M200 Pro microplate reader (TECAN, Switzerland). The turbidimetry (that increases with mycelial growth) was measured at 492 nm in each well [33]. Inhibition of spore germination and growth in liquid medium (GIs) was then estimated. In this experiment, the OD of the fungal positive control is equal to 1.7. If GIs was lower than 0, the number is replaced by 0 and the strain is considered as a non-inhibitory strain.

### Wheat protection test

The protection assay of wheat by candidate strains against *F*. *graminearum Fg1* was implemented in a greenhouse (INRA GDEC, Clermont-Ferrand). A panel of 12 strains was selected based on their *in vitro* efficacy against *Fg1*. In addition, 2 model biocontrol PGPR strains, *Pseudomonas kilonensis* F113 and *Bacillus amyloliquefaciens* AB42 (provided by the company BIOVITIS) were included in the greenhouse experiment. For each condition, 50 seeds of Chinese spring wheat cultivar Sumai 3 were distributed in 10 pots (12 x 10 x 10 cm) filled with 250 g of compost and 5 plants per pot. For each *in vivo* test, bacteria were prepared from TSB cultures grown over 24 hours at an optical density at 600 nm (OD_600nm_) of 1 (*i*.*e*. 10^8^ cells/mL) in sterile water. Bacteria were inoculated into each seed (10^7^ cells/seed) with 100 μL of prepared cultures. After 3 days, *Fg1* spores (10^5^ spores/seed) were added. Plants were watered every 3 days in order to keep soil water content close to 40% of Water Holding Capacity. After 45 days of culture, the experiment was stopped and the plants harvested. Different measurements were performed: (i) the chlorophyll rate of each wheat plant consisting of three measures performed on the last formed leaf using a SPAD 502 plus device (Minolta Camera Co., Osaka, Japan); (ii) the length (measured in cm) of the wheat strands; (iii) the dry weight of shoots; (iv) the disease symptoms of crown-rot on each wheat collar using a notation index ranging from 0 (no symptoms) to 3 (high symptoms); (v) the number of ears for each plant; (vi) dry weight of ears of each plant. For disease symptoms, the notation index was recorded as follow: 0 = no necrosis observed, 1 = necrosis observed on one sheet, 2 = necrosis observed one two sheets and 3 = necrosis observed on the whole collar. In addition, two different controls were also performed: (i) as a positive control, wheat was inoculated with only the pathogen spore suspension, and (ii) as a negative control, un-inoculated wheat was used.

### Statistical analyses

All statistical analyses were performed using R software. PCA analyses were performed to compared plant growth parameters and symptoms between treatments. Comparison of means was made using Kruskal-Wallis tests (*P*<0.05).

## Results

### Composition of the wheat-rhizosphere bacteria library

**Fig 1:**
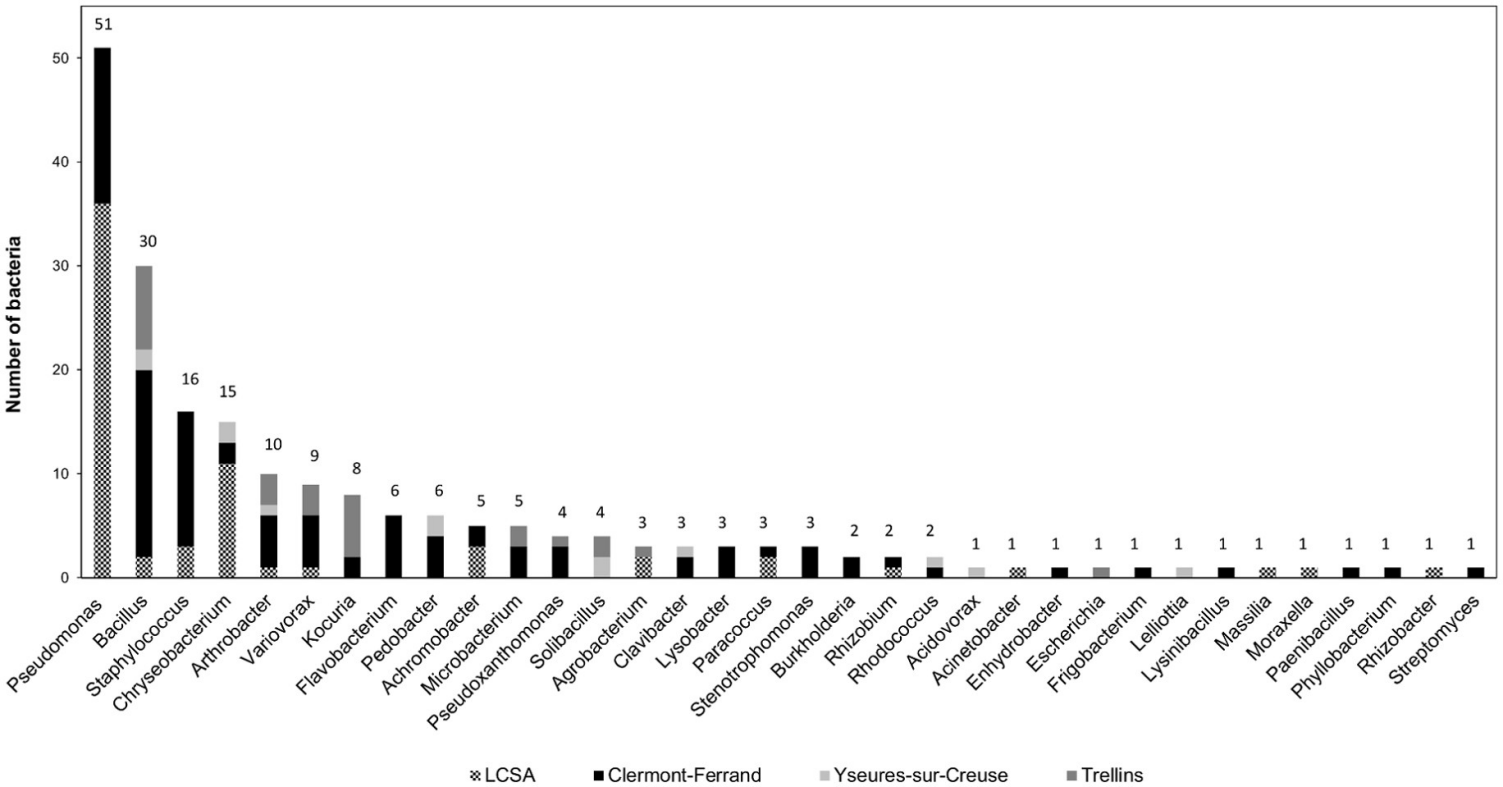
Histogram describing the composition of the wheat-rhizosphere soil bacterial library. The numbers above the bars refer to the number of isolates per genus recovered in all soils (Clermont-Ferrand, LCSA, Trelins and Yzeures-sur-Creuse).

Two hundred and three cultivable strains were isolated from the 4 soils by dilution-spreading. Two reference biocontrol strains were added: *P*. *kilonensis* F113 and *B*. *amyloliquefaciens* AB42 known to have antagonistic effects on fungi. These 205 strains are distributed among 34 different bacterial genera (Fig 1). The most represented genera are *Pseudomonas*, *Bacillus*, *Staphylococcus* and *Chryseobacterium* with respectively 51, 30, 16 and 15 strains per genus. The genera belong to 7 bacterial classes, distributed as follows: 32.5% of Gammaproteobacteria, 25.6% of Bacilli, 14.8% of Actinobacteria, 10.3% of Flavobacteria, 9.4% of Betaproteobacteria, 4.4% of Alphaproteobacteria, and 3.0% of Sphingobacteria. The sampling effort was not similar between the soils with, from the highest to the lowest: Clermont-Ferrand 97 strains, LCSA 66 strains, Trelins 27 strains and Yzeures-sur-Creuse 13 strains isolated. Accordingly, the greatest diversity of bacterial genera was obtained from isolates of Clermont-Ferrand soil with 25 different genera, followed by LCSA soil with 14 genera, then Trelins and Yzeures-sur-Creuse soils with 9 genera (Fig 1). The smaller sampling in the two latter soils, however, allowed us to increase the global diversity of the library, providing bacterial genera absent from the 2 other rhizosphere soils (Fig 1): *Acidovorax*, *Escherichia*, *Lelliottia*, and *Solibacillus*. Some genera with multiple representatives were present in only one particular soil: *Flavobacterium* (n = 6), *Lysobacter* (n = 3), *Stenotrophomonas* (n = 3), and *Burkholderia* (n = 2) in the Clermont soil. Only two genera, *Bacillus* and *Arthrobacter* were found in the 4 soils, and 2 genera are found in 3 soils: *Chryseobacterium* and *Variovorax*. Overall, eighteen most abundant genera (i.e. genera with more than 3 representatives per genus in the library) were present in the library.

### *Fg1* mycelial growth inhibition on solid medium

**Fig 2:**
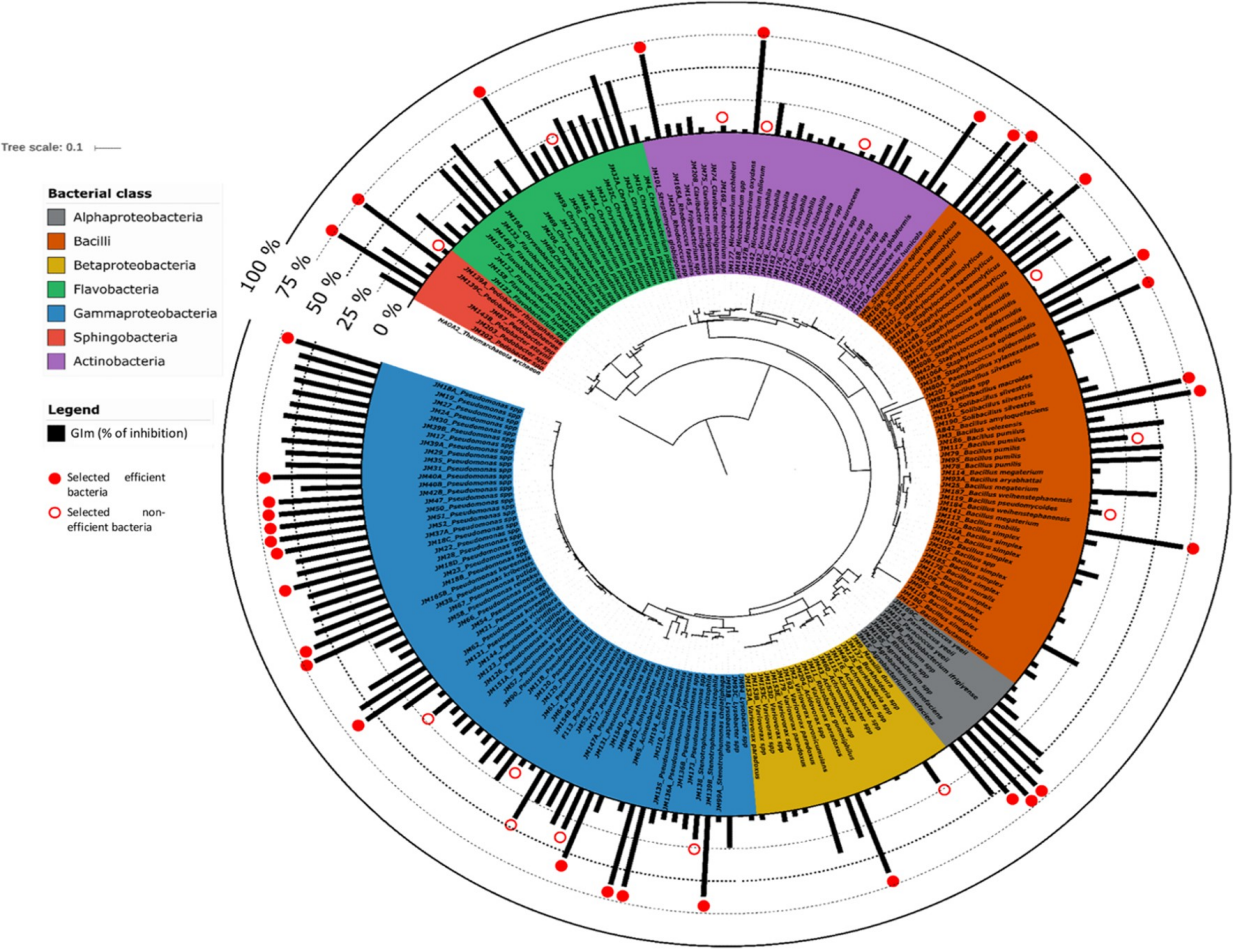
Mycelium growth inhibition activity (GIm) for the 205 bacterial isolates of the library according to their *rrs* taxonomical affiliation. The phylogenetic tree was inferred using the Neighbour-Joining method. GIm values (black bars) were reported on the *rrs* tree. The red solid circles indicate effective *Fg1*-antagonist bacteria, while empty red circles indicate non-effective *Fg1*-antagonist bacteria that were selected for the spore germination inhibition test. The tree was rooted with the *rrs* of the archaebacteria *Thaumarchaeota archaeon* NAOA2.

All isolates (205) were screened for their ability to inhibit the mycelial growth of *F*. *graminearum Fg1* on Petri dishes. GIm of each bacteria was reported on the phylogenetic tree of *rrs* gene sequences (Fig 2). Arbitrarily, a 50% GIm threshold was chosen to consider a strain as an inhibitory potential strain. A total of 61 strains obtained a GIm greater than or equal to 50%.

The distribution of the GIm values is heterogeneous among the bacterial genera, and none of the genus contained only effective inhibitory strains. While for some genera, such as *Clavibacter*, *Agrobacterium* or *Pseudoxanthomas*, GIm varied little, for other genera, such as *Bacillus*, *Pseudomonas*, *Chryseobacterium*, there was a great heterogeneity regarding their fungal inhibition ability (S2 Fig). For example, the genus *Bacillus* shared an average GIm of about 20% but half of the strains had a GIm of less than 10%. This means that some strains had a strong *in vitro* inhibitory capacity. However, at the genus level, the overall capacity of the genus was quite limited. In addition, the genus *Bacillus* contained the strain with the highest GIm (strain JM3). The GIm of *Achromobacter* strains (n = 5) was around 0%. In the most represented genera, *Pseudomonas* (n = 51) and *Staphylococcus* (n = 16), 55% of the strains share a GIm higher than 50%.

The influence of the soil origin of strains on their ability to inhibit the mycelial growth of *Fg1* was then analyzed (S3 Fig). When focusing on the genus *Bacillus*, the strains from the LCSA rhizosphere soil had GIm values much higher than the *Bacillus* strains present from the other soils (about 60% in LCSA, below 20% for the soil from Clermont-Ferrand and below 10% for the soils from Trelins and Yzeures-sur-Creuse). Results for the genus *Chryseobacterium* are opposite: the strains isolated from the Clermont-Ferrand rhizosphere soil are much more efficient, with an average GIm of about 75% than strains isolated from soils of LCSA (average GIm of 30%), and Yzeures-sur-Creuse (average GIm of 45%). Overall, the strains isolated from the LCSA soil seem to be more effective in the inhibition of the fungus than those isolated from the 3 other soils. Indeed, for the genera *Bacillus*, *Pseudomonas*, *Paracoccus* and *Staphylococcus*, the average GIm values are more important in LCSA soil than in the other soils. However, the Clermont-Ferrand soil contains strains from 8 different genera with GIm of at least 60% while this is only the case for 4 genera in the soil of LCSA, 2 genera in the soil of Trelins, and 1 genus in the soil of Yzeures-sur-Creuse (S3 Fig).

### *Fg1* spore germination and mycelial growth inhibition in liquid medium

From the different results obtained during solid-state inhibition tests, 33 very effective strains showing a GIm higher than 50% from 14 different genera were selected (i.e. *Chryseobacterium* (n = 1), *Pedobacter* (n = 2), *Arthrobacter* (n = 1), *Rhodococcus* (n = 1), *Microbacterium* (n = 1), *Bacillus* (n = 3), *Staphylococcus* (n = 5), *Rhizobium* (n = 1), *Agrobacterium* (n = 2), *Stenotrophomonas* (n = 1), *Lelliottia* (n = 1), *Escherichia* (n = 1), *Pseudomonas* (n = 12) and *Variovorax* (n = 1)). In addition, we also selected 14 strains with little or no-inhibitory activity against *Fg1* and belonging to 8 common genera (i.e. *Chryseobacterium* (n = 1), *Pedobacter* (n = 1), *Arthrobacter* (n = 1), *Microbacterium* (n = 2), *Bacillus* (n = 2), *Staphylococcus* (n = 2), *Stenotrophomonas* (n = 1), and *Pseudomonas* (n = 4)) in order to avoid the mis-selection of relevant bacterial candidates expressing a distinct mechanism of action on *F*. *graminearum*, such as ability to inhibit asexual spore germination.

**Fig 3:**
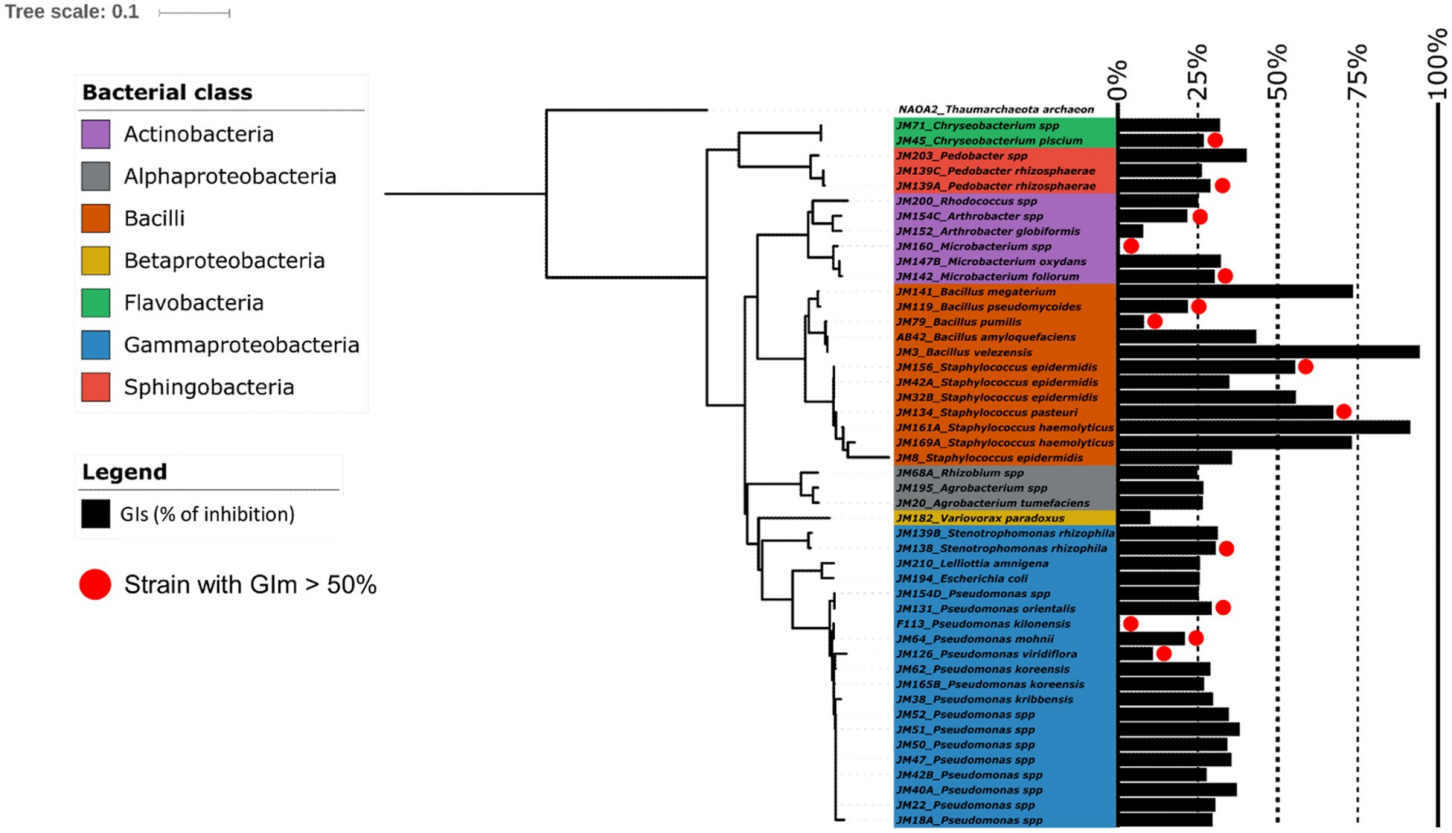
Spore germination inhibition activity (GIs) for the 47 strains selected after the first inhibition test, according to their rrs-based taxonomical affiliation. The phylogenetic tree was inferred with the Neighbor-Joining method and GIs were reported on this tree (black bars). The red solid circles indicate bacteria that were inhibitory efficient strains (GIm > 50%) when evaluating their ability to inhibit the Fg1 mycelium growth in the first screening test.

A total of 45 strains from the library and 2 reference strains were selected for this evaluation; they were divided into 14 different genera (Fig 3, S4 Fig). As the bacterial mechanisms involved for the inhibition of spore germination may differ from those involved in the inhibition of mycelial growth, our broad strain selection may allow us to select new potential effective strains unrevealed *via* the inhibition growth test on solid medium. Two genera shared the highest average GIs values of about 50%: *Bacillus* and *Staphylococcus*. They included the two most effective spore germination inhibiting bacteria, strains JM3 (*Bacillus* with a GIs of 94%) and JM161A (*Staphylococcus* with a GIs of 91%). However, a great variability level of GIs was observed in these two genera, with data ranging from 94% to 8% for *Bacillus*, and from 91% to 25% for *Staphylococcus* (S4 Fig). In other genera, except *Arthrobacter* and *Variovorax*, for which average GIs were respectively equal to 14% and 10%, the average GIs was equal to or higher than 25% (S4 Fig). Seven strains out of 47 have a GIs higher than 50% (5 *Staphylococcus* and 2 *Bacillus*).

**Fig 4:**
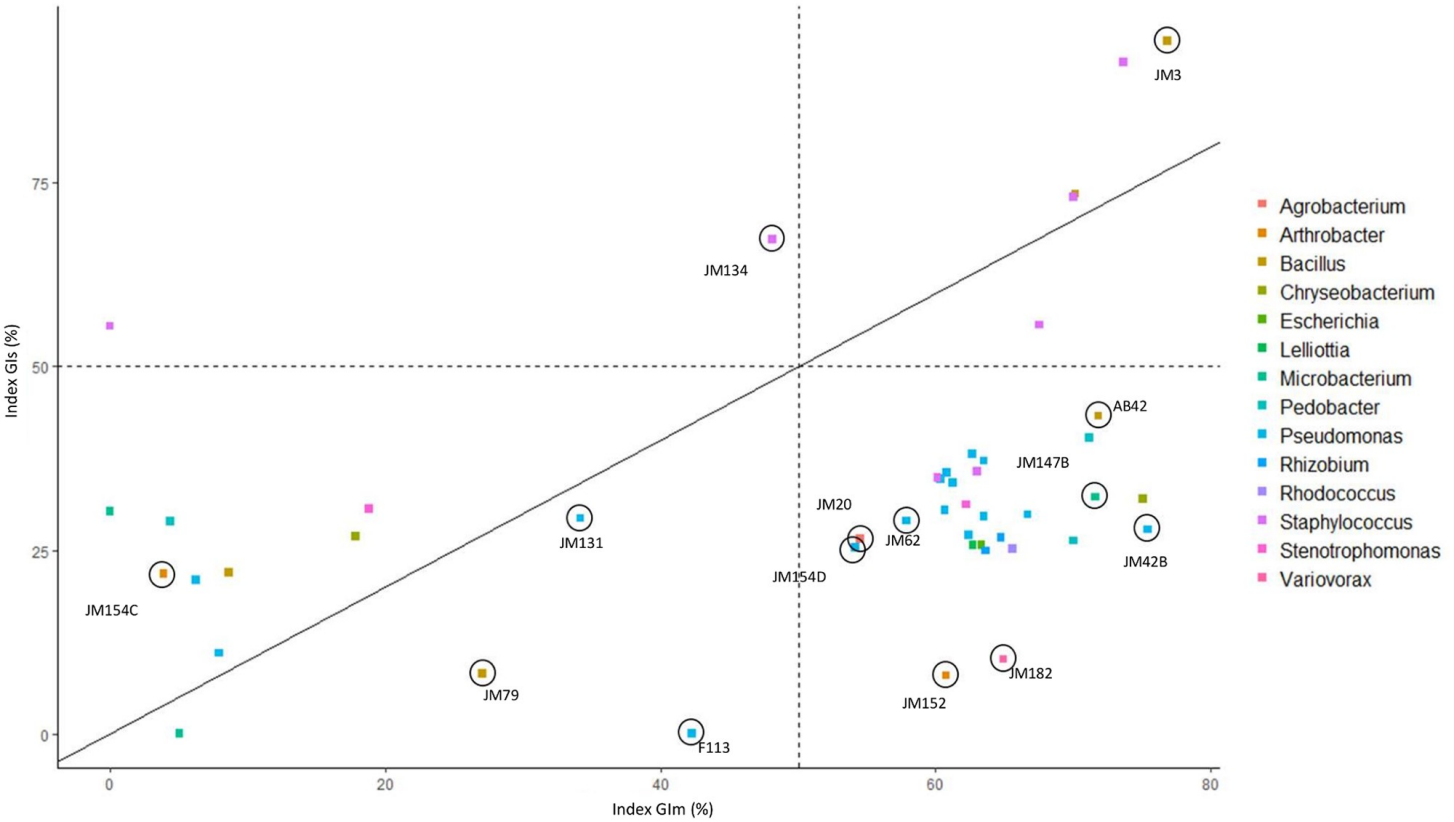
Selection of strains for in planta experiments according to their GIm and GIs. The solid line represents GIs equal to GIm. Bacteria more efficient for inhibiting the spore germination are located above this line, while bacteria more efficient for inhibiting the mycelium growth are located below this line. Dashed lines represent values of GIm and GIs equal to 50%. Bacteria selected for the evaluation of their plant protection activity against *F*. *graminearum Fg1* are surrounded by dark circles.

For the 47 strains, the results of their GIm and GIs values were combined (Fig 4) and grouped into four distinct classes: class 1 contains strains with GIm and GIs higher than 50%, classes 2 and 3 respectively contain strains with a GIm higher than 50% and a GIs lower than 50% and *vice-versa*, and class 4 GIm and GIs lower than 50%. Fourteen strains belonging to each of these classes (1 strain in class 1 among 5, 8 in class 2 among 28, 1 in class 2 among 2 and 4 in class 4 among 12) were then selected for the *in vivo* pot experiments based on their *Fg1* inhibition activities and taxonomic affiliation. These 14 strains belong to 7 distinct genera: *Pseudomonas* (n = 5; strains JM131, JM154D, JM42B, JM62, F113), *Bacillus* (n = 3; strains AB42, JM79, JM3), *Staphylococcus* (n = 1; strain JM134), *Microbacterium* (n = 1; strain JM147B), *Arthrobacter* (n = 2; strains JM152, JM154C), *Agrobacterium* (n = 1; strain JM20), and *Variovorax* (n = 1; strain JM182).

### Wheat protection test

**Fig 5:**
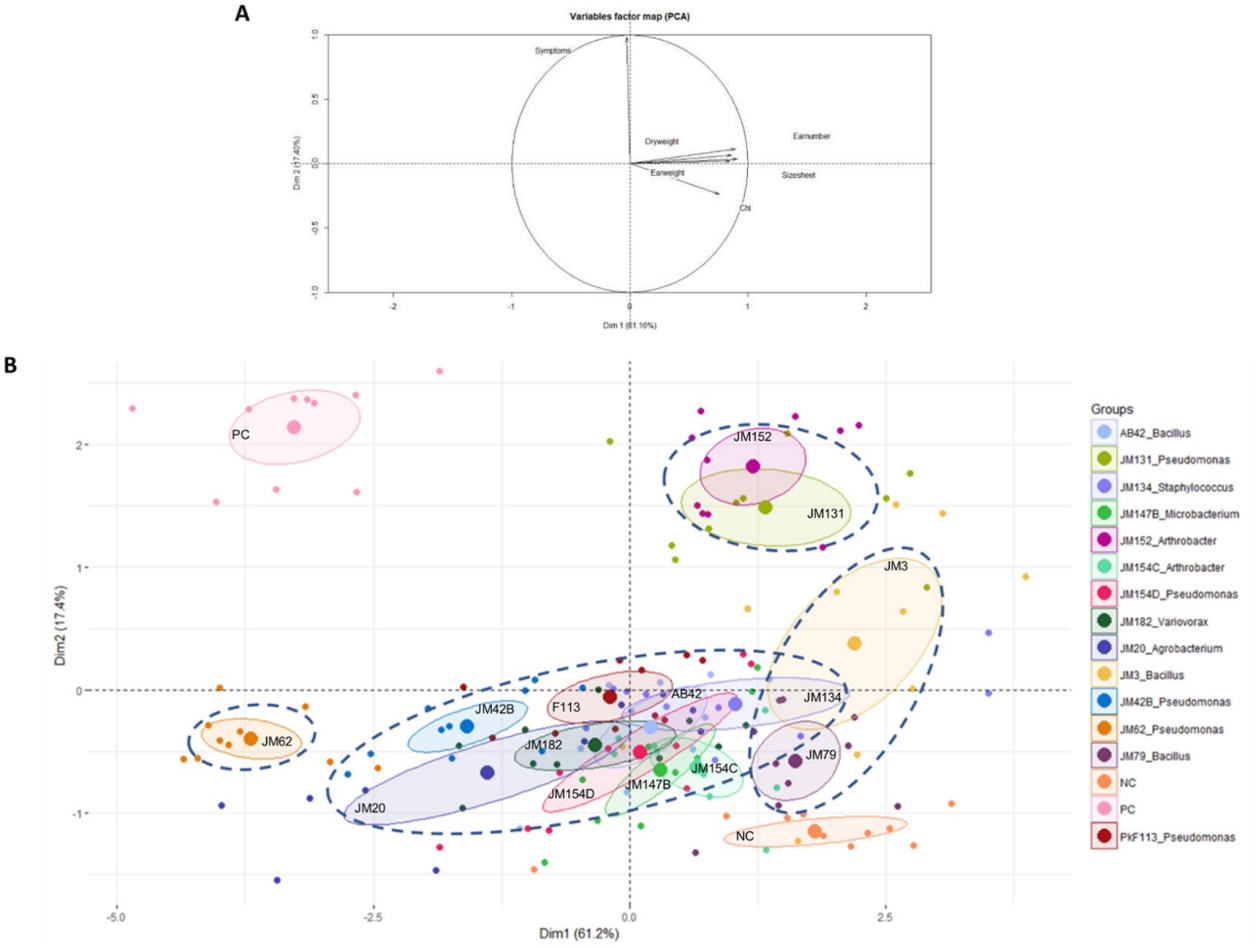
Discriminant principal component analysis (PCA) of plant growth and disease symptom parameters of wheat plants inoculated with each of the 14 bacterial strains in presence of *F*. *graminearum Fg1*. (A) Correlation circle between recorded plant parameters. (B) PCA (first two axes) showing groups of strains (dashed line circles) inducing similar responses on plants. NC: negative control (un-inoculated wheat plants). PC: positive control (wheat plants inoculated with *Fg1* only).

After 45 days of culture, 6 different plant parameters were recorded on wheat and analyzed together using PCA analysis (Fig 5A and 5B). Significant differences were observed between the negative (NC) and the positive (PC) controls with percentages of the first axis reaching 61.2% and the second axis, 17.4%. This means that the pathogen inoculation modified various measured plant parameters and significantly affected the physiology of the wheat samples. The distribution of the data is explained by two main groups of factors: plant growth parameters on one hand (*i*.*e*. length and dry weight of wheat strands, chlorophyll content, number and weight of wheat ears) and the intensity of disease symptoms (crown rot) on the other hand (Fig 5A).

The data were divided into 4 groups (Fig 5B). The first group was composed of strains JM79 and JM3 from *Bacillus* genus, which are close to the negative control (NC; non-inoculated plants), showing a similar growth as healthy plants (same position on PCA’s axis 1 and no significant difference for all growth traits) and reduced disease symptoms (Table 1, S5 Fig). The second group is composed of strains JM134 (*Staphylococcus*), JM154C (*Arthrobacter*), AB42 (*Bacillus*), JM147B (*Microbacterium*), JM154D, F113 and JM42B (*Pseudomonas*), JM182 (*Variovorax*), and JM20 (*Agrobacterium*). These strains share common data for growth parameters (axis 1) that are lower than the negative control but significantly higher than the positive control (PC; inoculated with the pathogen) (Fig 5B, Table 1, S5 Fig). For this cluster of strains, weak disease symptoms were observed. A third group was composed of strains JM131 and JM152 (Fig 5B, Table 1, S5 Fig). These strains exhibited growth parameters as high as those shared by the NC but with disease symptoms as high as the positive control. The last group included the strain JM62. Unlike the previous groups, this strain presented reduced growth (*i*.*e*. similar growth parameters as the positive control) but weak disease symptoms.

**Table 1:** Statistical comparison of wheat growth and symptom traits between inoculation treatments.

PCA groups	Strains	Genus	Chlorophyll	Shoot dry weight	Ear Number	Ear weight	Strand length	Symptoms
**NC**	NC	-	ab^$^	ab	ab	a	a	g
**Group 1**	JM79	*Bacillus*	a	ab	abcd	a	ab	ef
	JM3	*Bacillus*	a	a	ab	ab	a	bcd
**Group 2**	JM134	*Staphylococcus*	abcde	abc	ab	abc	bcde	cde
	JM154C	*Arthrobacter*	abcd	abc	bcde	abcd	abc	ef
	AB42	*Bacillus*	efg	bcde	abc	ab	cdef	def
	JM147B	*Microbacterium*	abcde	bcde	abcd	abcd	bcde	fg
	JM154D	*Pseudomonas*	cdefg	bc	bcde	abcd	abcd	ef
	PkF113	*Pseudomonas*	gh	abc	cdef	abcd	abcd	def
	JM42B	*Pseudomonas*	fgh	def	efg	de	def	def
	JM182	*Variovorax*	bcdef	cdef	defg	bcde	abcd	def
	JM20	*Agrobacterium*	defg	cdef	efg	cde	def	fg
**Group 3**	JM131	*Pseudomonas*	abcde	abc	ab	a	abc	abc
	JM152	*Arthrobacter*	abc	bcd	a	abc	abc	ab
**Group 4**	JM62	*Pseudomonas*	h	f	g	e	f	ef
**PC**	PC	-	h	ef	fg	e	ef	a

NC: negative control (un-inoculated wheat plants). PC: positive control (wheat plants inoculated with *Fg1* only).

^$^ Statistical differences between strains for each plant parameters are indicated with letters a to h. (ANOVA and Tukey’s HSD test, *P* < 0.05)

## Discussion

*F*. *graminearum*, a telluric fungus, particularly present in contaminated plant debris, is the main causal agent of the Fusarium Head Blight disease (FHB) on small grain cereals. In our study, given our interest for protecting wheat against *F*. *graminearum*, our library was focused on bacteria growing in the rhizosphere of wheat.

The bacterial genera most commonly found in plant protection studies are the genera *Pseudomonas*, and *Bacillus*. In our study, a more global approach was implemented based on the isolation of culturable bacteria on a generalist agar 1/10-diluted TSA medium, without targeting any distinct genus. Some un-culturable bacteria present in the rhizosphere could of course not be targeted by this protocol, knowing that less than 1% of soil bacteria are considered culturable. Given that un-culturable bacteria can hardly be exploited to develop biocontrol agents, this protocol bias may be acceptable. Another bias in selecting soil bacteria on 1/10-diluted TSA media is also linked to the fact that most abundant strains in the starting sample are more likely to be isolated, as well as the most competitive strains. Obviously, a small part of the biodiversity present in our starting soil samples was explored. However, our strain library held 34 different bacterial genera, and the genera found in our study are consistent with previous studies performed on wheat rhizosphere soils [34, 35].

In particular, *Bacillus* and *Pseudomonas* were the two most abundant genera found in the library (Fig 1). This result is consistent with data recorded in the literature as these genera grow easily on growth media and are abundant in rhizosphere soils [36–38]. More surprisingly, the genus *Staphylococcus* is the 3^rd^ most represented genus. *Staphylococcus* is well documented in the literature, but in the domain of human health, where *Staphylococcus aureus* is known as one species involved in severe nosocomial diseases. Given the fact that *Staphylococcus* strains are well-known as human commensal or pathogens, sparse information regarding the abundance of *Staphylococcus* populations in soil [37] and their use for agriculture purposes is available. However, several studies have shown that some *Staphylococcus* strains can harbor plant growth promoting properties such as IAA or siderophore production and Zn solubilization [38, 39]. The beneficial effects of certain *Staphylococcus* in agriculture, particularly on wheat, with significantly improved dry and wet plant biomasses, root length and shoot length were also reported [40]. The genus *Staphylococcus* was thus kept in our strain library to study its potential effect on *F*. *graminearum Fg1*.

The strains were then selected according to their ability to inhibit the mycelial growth of the fungus on agar plate, and to inhibit the germination of *F*. *graminearum* spore and mycelial growth in a liquid medium. Both tests were performed in order to select strains that can inhibit *Fg1* during different steps of its life cycle. Indeed the pathogen can be found in soils either as mycelium or spores (sexual and asexual) in plant debris [41].

Dual-culture agar tests were commonly implemented for identifying bacteria potentially usable as biocontrol agents against fungal or bacterial plant pathogens [42]. Given the effectiveness of their implementation, dual-plate tests are suitable as a first screening step. Using dual-culture growth inhibition tests on plate and selecting a threshold GIm higher than 50%, 61 strains of the library, belonging to 14 different genera, were considered as potentially interesting biocontrol strains. These 61 strains account for 30% of the strains efficient against *F*. *graminearum* in the library, a percentage in line with different studies [42–45], where between 10 and 50% of bacteria from libraries were found efficient in combating fungal pathogens.

We have therefore been able to highlight the diversity of bacterial genera with *in vitro* fungal antagonistic activity. For spore inhibition microplate tests, we kept 33 strains of interest with GIm higher than 50%, including the top 30. The originality of our approach was to select, in addition, strains that did not give promising results on the dual-culture plate test. Among the 14 latter strains, GIm ranges from 0% to 48%. This choice allowed us to highlight strains which were not effective in the growth inhibition test on agar plate but which proved to be efficient to inhibit spore germination and mycelium growth in liquid medium *via* the production of antifungal compounds in culture supernatants. Some strains like JM134 or JM156, the latter belonging to the *Staphylococcus* genera, show better inhibition activity on spore germination than on mycelium growth on solid medium. These results highlight that different antagonism mechanisms exist in bacteria to achieve the inhibition of mycelium and spore germination of *F*. *graminearum*. It demonstrates that less studied antagonist effects targeting spore germination might be a promising way to discover new bacterial mechanisms and molecules involved in *F*. *graminearum* inhibition. Recently, it has been shown that phenazines produced by some *Pseudomonas* PGPR can directly affect the activity of histone acetyltransferases in *F*. *graminearum* leading to deregulation of histone acetylation, and consequently, impairment of its growth and virulence [46]. Indeed, the original microplate test developed in the present work allows us to highlight the potential role of other secondary metabolites released by strains in supernatant culture, inhibiting the spore germination or liquid growth of *F*. *graminearum*. Of the 47 selected bacteria tested in microplate, 7 strains obtained GIs greater than 50%. These strains belong to only 2 genera: *Bacillus* and *Staphylococcus*. The secondary metabolites of *Bacillus* are well described with several classes of lipopeptides known to inhibit various plant pathogens [47]. Due to the lack of studies on the use of *Staphylococcus* genus for bioinoculant purposes, very few metabolites are known in the plant-*Staphylococcus* interaction. Some studies have already explored the phytostimulation effects of the *Staphylococcus* genus, evidencing their ability to synthetize auxin, produce siderophores or to solubilize phosphorus [39, 40], but to our knowledge, no study has reported any plant protection potential against soil-borne fungal pathogens of cereals.

In our wheat protection experiment, strains were selected to maximize the diversity of the strains at the taxonomic level and the diversity of their action mechanisms, through a selection of strains effective in the two *in vitro* tests or, on the contrary, with weak *in vitro* activities. In addition to testing *Pseudomonas* and *Bacillus* strains, we thus kept for the *in planta* test other genera like *Staphylococcus* but also *Arthrobacter*, *Variovorax* or *Microbacterium*. These have been included in some plant experiments to evaluate their plant growth stimulation effects, but in few plant protection assays [48–50]. In order to offer a wider panel of biocontrol products to farmers, focusing on other bacterial genera than *Bacillus* and *Pseudomonas*, could allow us to obtain strains that may have other antagonistic modes of action than the well-known synthesis of cyclic lipopeptides and polyketides [12, 47].

The *in planta* experiments in greenhouse have shown that the strains expressing the best activities *in vitro* are not always the strains showing the best results *in vivo* and *vice versa*, as recently reported by Comby et al. [51]. Indeed, the *Bacillus* sp. strain JM79 gave the best plant growth stimulation and plant protection results, but very limited antagonistic activity *in vitro*.

While most plant protection studies aim to select bacteria with high fungal antagonistic potential *in vitro*, our study highlights that it is not a *sine qua non* condition for a successful plant protective activity against fungal pathogen. Furthermore, other mechanisms than the ability to inhibit the growth of the pathogen can contribute *in planta* to a better resistance of the plant to the disease. First, some bacteria are able to elicit plant induced systemic resistance (ISR) [52, 14]. Fluorescent *Pseudomonas* and *Bacillus* PGPR are well known as being able to induce ISR responses by producing secondary metabolites like 2,4-diacetlyphloroglucinol, volatile organic compounds or cyclic lipopeptides, and as a way to protect the plant, including wheat [47, 52–54]. Through the induction of still imprecisely described hormonal regulation networks, PGPR bacteria can elicit plant defense responses fast when a pathogen attacks the plant [55]. These faster defense responses will lead to a better resistance of the plant to its aggressor. In addition, some PGPR are known to enhance the bioavailability of essential elements to plant, such as nitrogen, phosphorus, potassium, but also iron and other rare but essential mineral elements [11]. When a plant is not suffering from nutrition limitation, it can express its development program faster, allowing its best resistance to pathogen attack, especially at key steps like seed germination and seedling growth [56]. In addition, PGPR can also play a role in resistance to abiotic stress, such as drought, tolerance to salt, heavy metal [57, 58]. Abiotic stresses can make the plant more vulnerable to pathogen attack because biotic and abiotic stresses simultaneously induce various plant responses that can have negative impacts on the growth [59]. Nevertheless, some studies highlight the positive effect of some abiotic stress in resistance to biotic stress through elicitation of some joint metabolic pathways [60–62]. The complexity of soil interactions between prokaryote and eukaryote organisms makes selection of effective strains difficult and tricky. These considerations underline the importance to broaden studied models and scales of studies. Combining bacteria with different modes of actions in inoculant consortia might be an interesting way to circumvent the difficulty of finding strains with good efficacy whatever the tested soils, crops and agrosystems [63]. The plant microbiome itself could contribute to plant health, particularly through the modulation of ISR responses, the direct inhibition of pathogen growth in the rhizosphere such as that occurring in disease-suppressive soils [64]. Naturally stimulating the biocontrol activities of this beneficial microbiome through the use of appropriate crop accessions and suitable cultural practices is a way forward.

Our study highlighted various ways to achieve plant protection with biocontrol bacteria. Interestingly, antagonist interactions *in vitro* may not always lead to successful protection *in planta* because there are a lot of biotic interactions in soil and with the plant that can lead to biocontrol agent failure [65, 66].

## Conclusion

Our work was performed in order to select microorganisms that can be used as biological control agents against Fusarium seedling blight. By conventional methods targeting cultivable microorganisms, we have demonstrated that combining *in vitro* and *in planta* protocols targeting different steps of the fungal pathogen life cycle (spore germination, mycelial growth and plant invasion) enabled us to refine the selection of effective biocontrol agents against *F*. *graminearum*. However, one should be aware that the *in vitro* selection of biocontrol agents based on fungal growth inhibition dual-plate tests can set apart potentially interesting biocontrol strains.

## Supporting information

S1 Fig GIm (%) description. A: description of the GIm used to define the efficacy of a strain against the mycelial growth of *F*. *graminearum* on PDA medium. For each strain, including the fungal control, an index I is calculated and used for calculating the mycelium growth inhibition on solid medium GIm. B: photographs illustrating 4 different levels of GIm.
(TIF)

S2 Fig Box plots of GIm (%) of isolates depending on the bacterial genus they belong to. The numbers in parenthesis refer to the number of isolates belonging to the indicated genera in the whole library. Only genera with more than 2 isolates were presented. Gray diamonds represent mean values for each genus. The bacterial genera were classified according to the class taxonomic level.
(TIF)

S3 Fig Box plots of GIm (%) of isolates depending on their genus and their soil origin. Only genera with more than 2 isolates were presented. Gray diamonds represent mean values for each genus.
(TIF)

S4 Fig Box plots of GIs (%) of 47 isolates depending on the bacterial genus they belong to. The numbers in parenthesis refer to the number of isolates per genus that were tested in the fungal growth inhibition test in liquid medium. Gray diamonds represent mean values for each genus.
(TIF)

S5 Fig Box plots of values for the different parameters recorded during the wheat protection experiments for the 14 tested strains. NC = Negative control. PC = Positive control. The measured parameters are: (A) the chlorophyll content expressed in arbitrary unit, (B) dry weight of shoot expressed in g, (C) number of ears by plant, (D) dry weight of ears expressed in g, (E) length of wheat strands expressed in cm, (F) symptoms expressed in arbitrary unit (according to the scale described in the Material and Method section). Gray diamonds represent means.
(TIF)
