# Supplementary figures and images for: Does in vitro selection of biocontrol agents guarantee success in planta? A study case of wheat protection against Fusarium seedling blight by soil bacteria
Source: PLoS One. 2019 Dec 5;14(12):e0225655. doi: 10.1371/journal.pone.0225655 (PMC6894788; doi:10.1371/journal.pone.0225655)

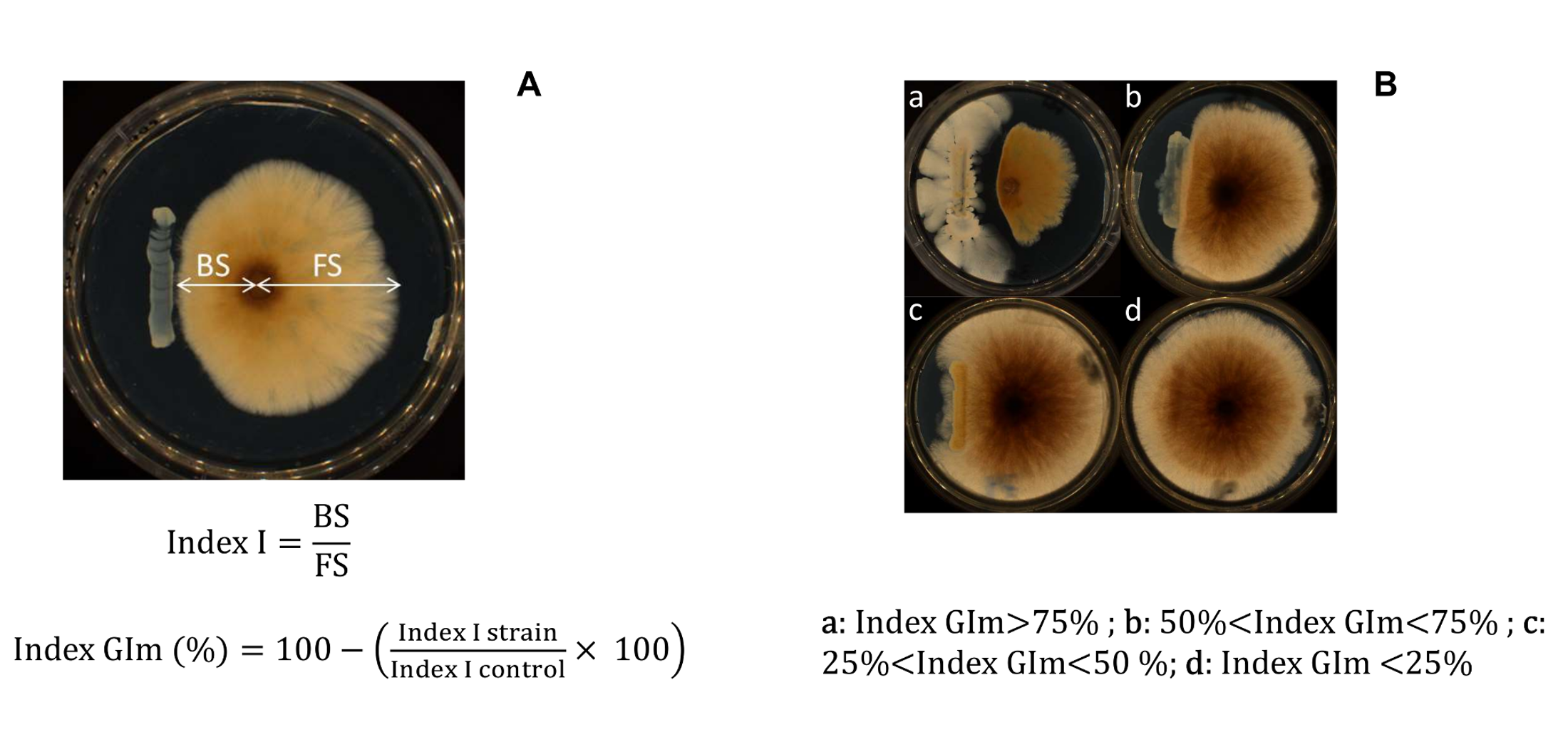

Supplement: S1 Fig — A: description of the GIm used to define the efficacy of a strain against the mycelial growth of F. graminearum on PDA medium. For each strain, including the fungal control, an index I is calculated and used for calculating the mycelium growth inhibition on solid medium GIm. B: photographs illustrating 4 different levels of GIm. (TIF) [file pone.0225655.s001.tif]

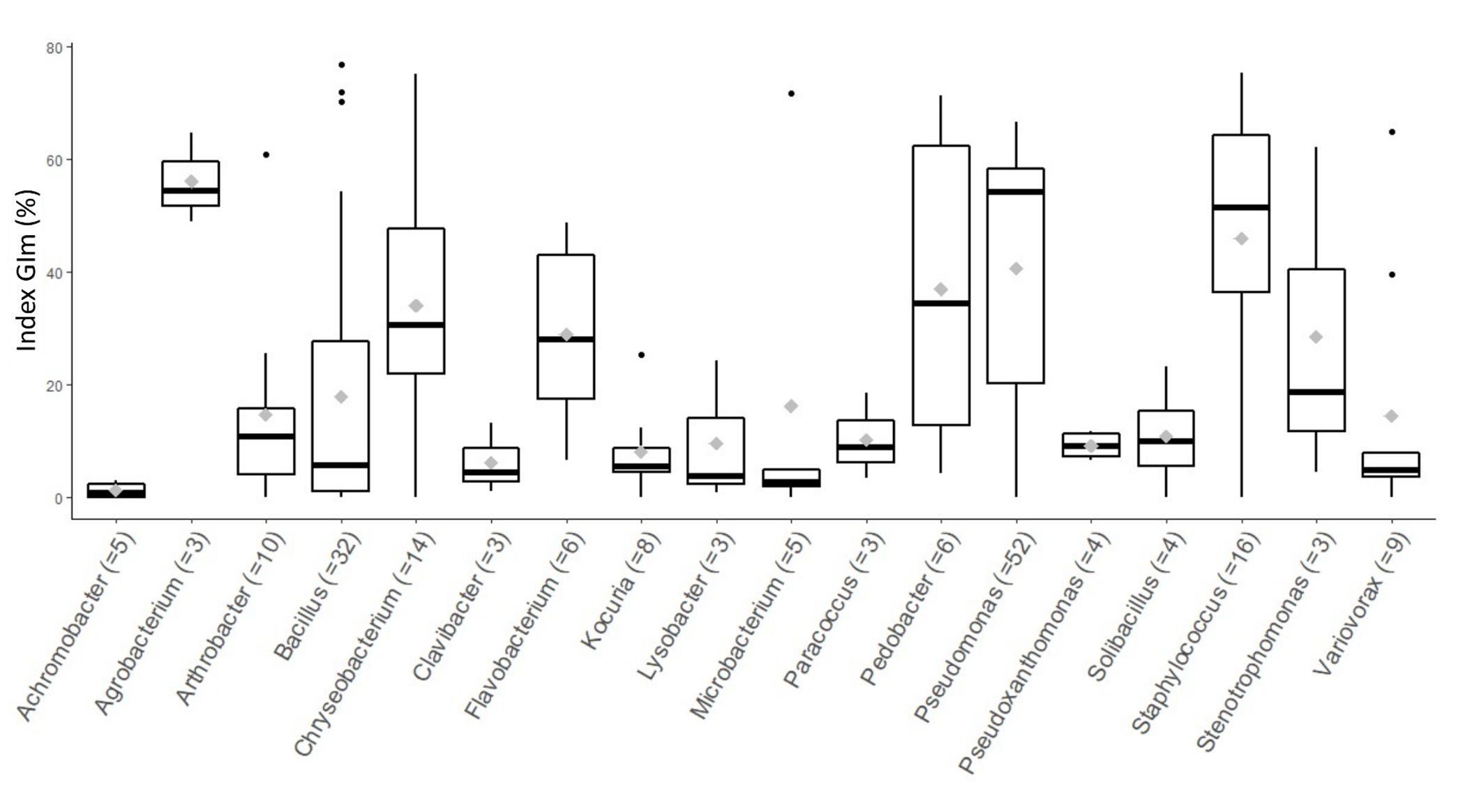

Supplement: S2 Fig — The numbers in parenthesis refer to the number of isolates belonging to the indicated genera in the whole library. Only genera with more than 2 isolates were presented. Gray diamonds represent mean values for each genus. The bacterial genera were classified according to the class taxonomic level. (TIF) [file pone.0225655.s002.tif]

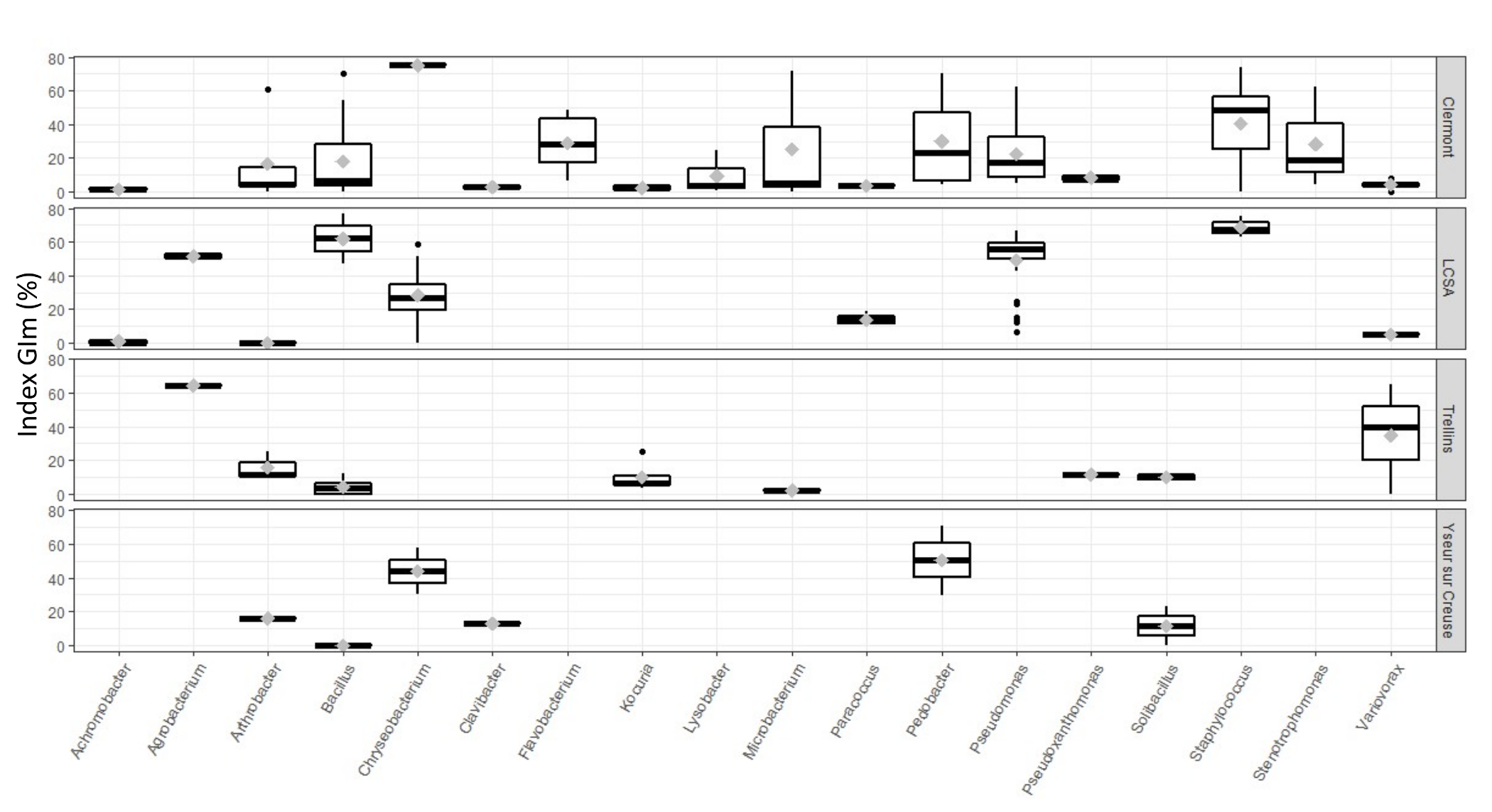

Supplement: S3 Fig — Only genera with more than 2 isolates were presented. Gray diamonds represent mean values for each genus. (TIF) [file pone.0225655.s003.tif]

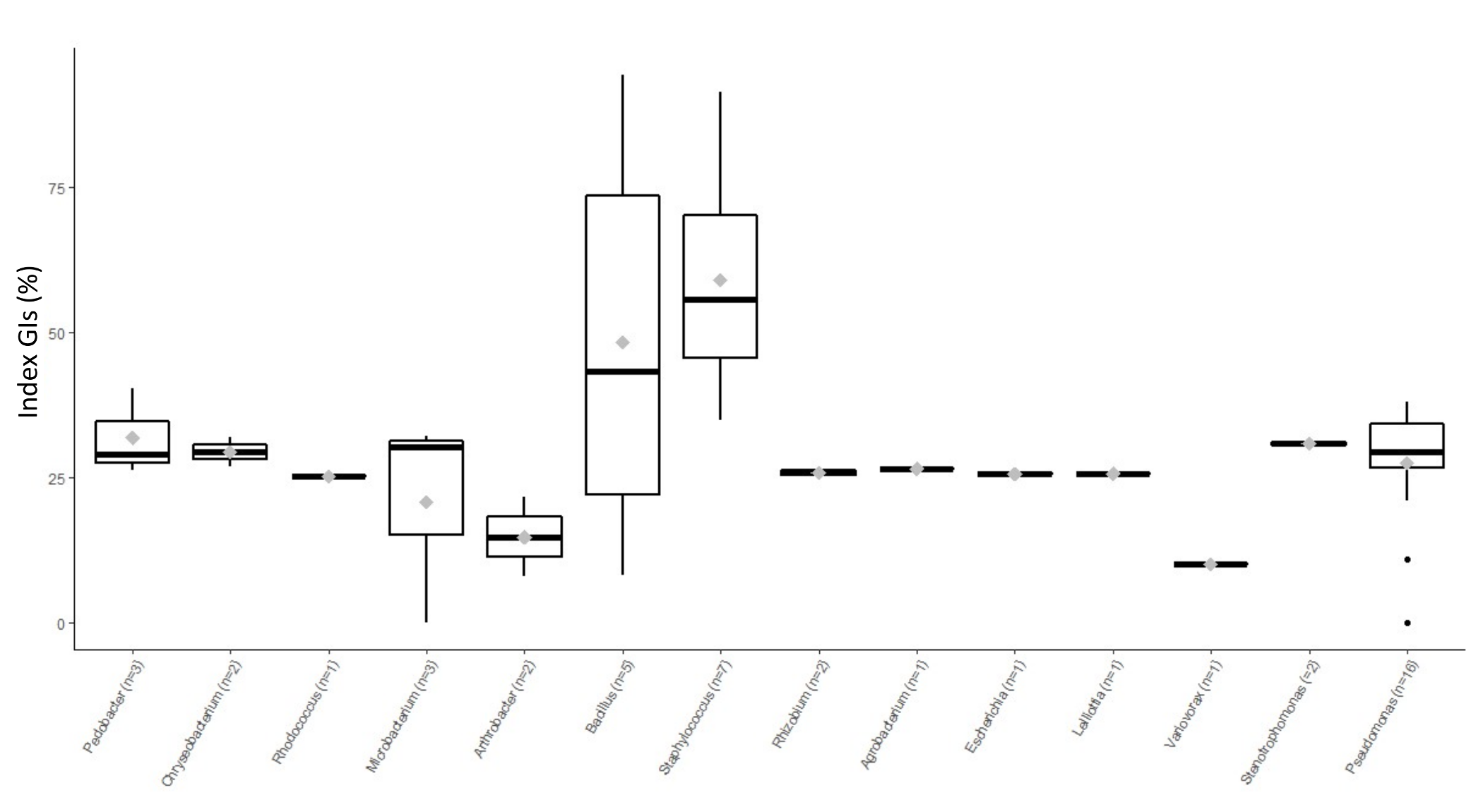

Supplement: S4 Fig — The numbers in parenthesis refer to the number of isolates per genus that were tested in the fungal growth inhibition test in liquid medium. Gray diamonds represent mean values for each genus. (TIF) [file pone.0225655.s004.tif]

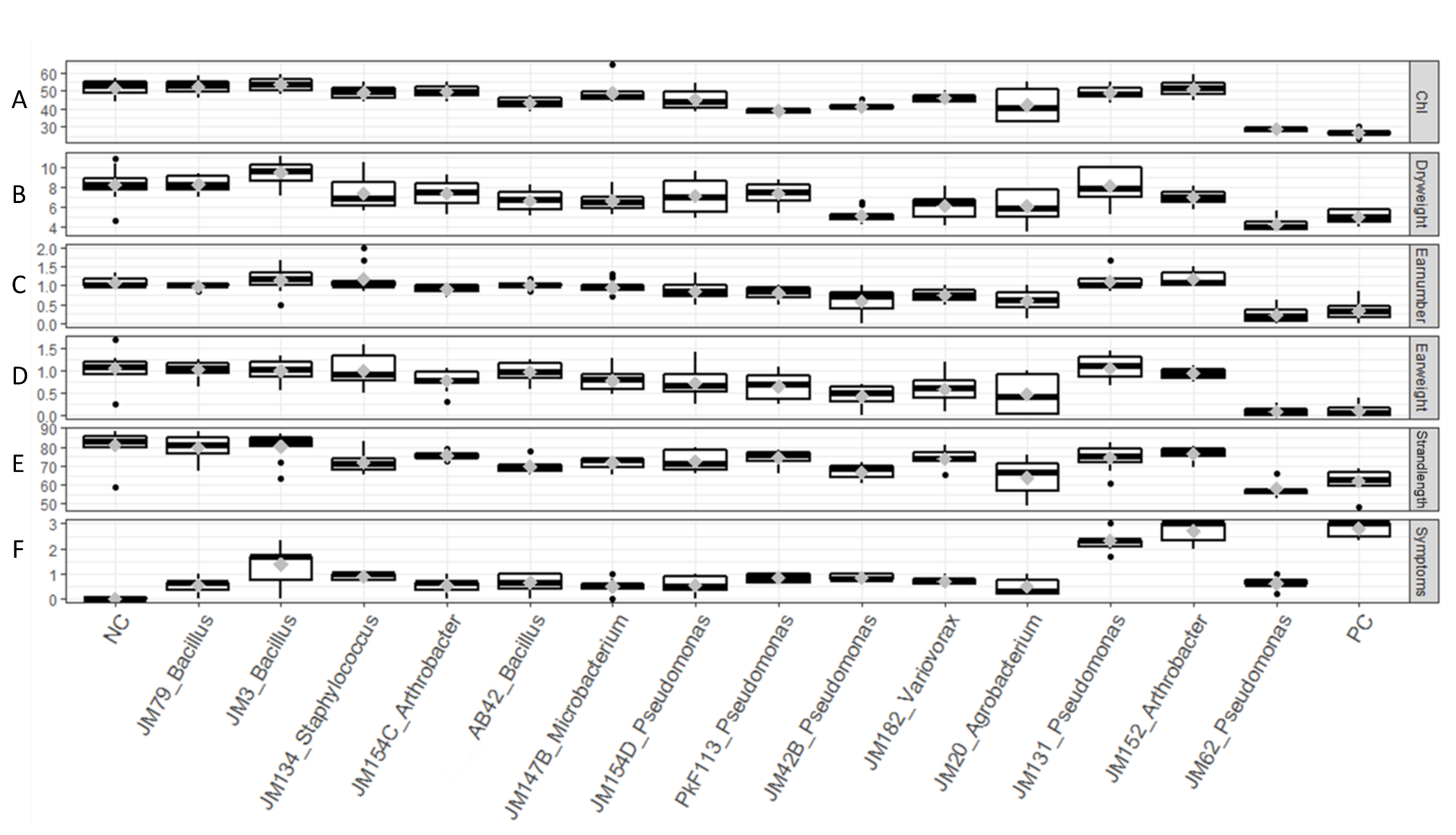

Supplement: S5 Fig — NC = Negative control. PC = Positive control. The measured parameters are: (A) the chlorophyll content expressed in arbitrary unit, (B) dry weight of shoot expressed in g, (C) number of ears by plant, (D) dry weight of ears expressed in g, (E) length of wheat strands expressed in cm, (F) symptoms expressed in arbitrary unit (according to the scale described in the Material and Method section). Gray diamonds represent means. (TIF) [file pone.0225655.s005.tif]
